# A Milestone in Cardiac Care: The Intra-Aortic Balloon Pump in Cardiac Surgery and Transplantation

**DOI:** 10.21470/1678-9741-2024-0991

**Published:** 2024-05-13

**Authors:** Alvaro Perazzo, Samuel Padovani Steffen, Fabio Antônio Gaiotto, Ronaldo Honorato Barros Santos, Fabio Biscegli Jatene, Roberto Lorusso

**Affiliations:** 1 Cardiovascular Surgery Division, Instituto do Coração (InCor) do Hospital das Clínicas da Faculdade de Medicina da Universidade de São Paulo, São Paulo, Brazil; 2 Heart Transplant Department, Instituto do Coração (InCor) do Hospital das Clínicas da Faculdade de Medicina da Universidade de São Paulo, São Paulo, Brazil; 3 Maastricht University Medical Centre, Department of Cardio-Thoracic Surgery, Maastricht, Netherlands; 4 Maastricht University, Cardiovascular Research Institute Maastricht, Maastricht, Netherlands

**Fig. 1:**
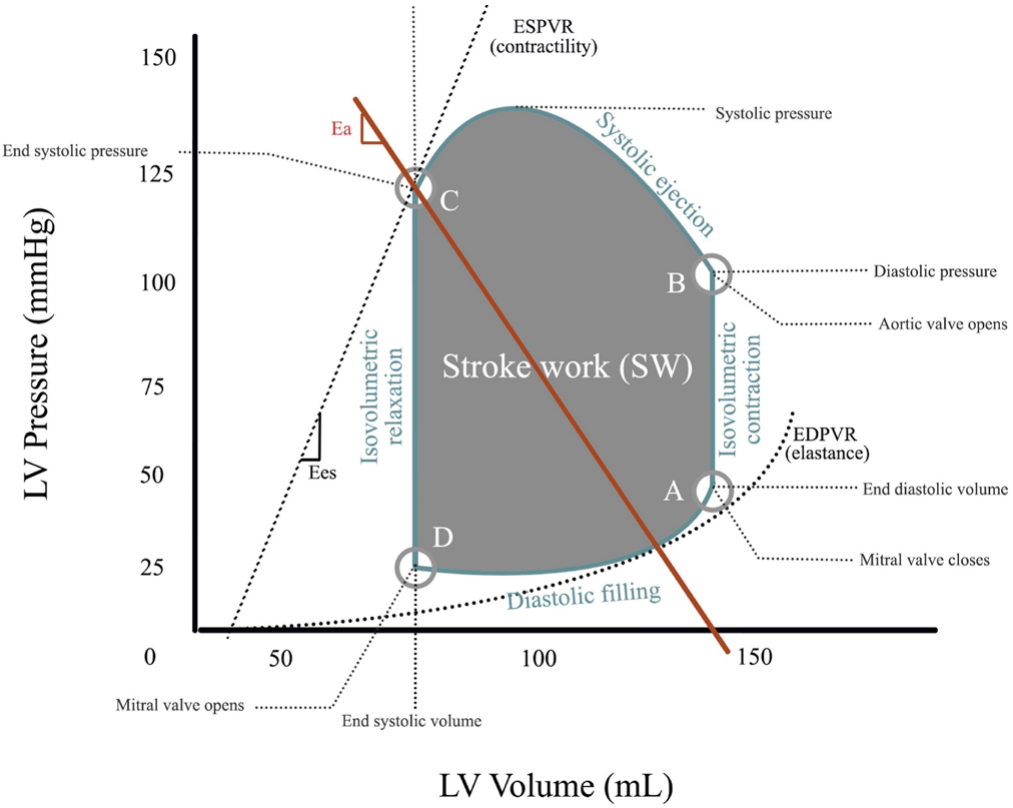
Normal cardiac physiology demonstrated by pressure-volume loop. This figure illustrates the normal cardiac physiology showing the relationships between volume and pressure in the left ventricle. The pressure-volume loop depicts the diastolic filling of the ventricle (D to A), early systole and isovolumetric contraction (A to B), systolic ejection (B to C), and ventricular isovolumetric relaxation (C to D). Key points include the opening and closure of the semilunar and atrioventricular valves during different phases of the cardiac cycle. Ea=afterload; EDPVR=end-diastolic pressure-volume relationship; Ees=end-systolic elastance; ESPVR=end-systolic pressure-volume relationship; LV=left ventricular.

*Source: Gillespie LE, Lane BH, Shaw CR, Gorder K, Grisoli A, Lavallee M, et al. The Intra-aortic Balloon Pump: A Focused Review of Physiology, Transport Logistics, Mechanics, and Complications. J Card Surg. 2024 Feb 18;101337. doi: 10.1016/j.jscai.2024.101337*

For over five decades, the intra-aortic balloon pump (IABP) has been a cornerstone as a mechanical cardio-circulatory support (MCS) in cardiac surgery and heart transplantation, especially in developing nations. This device, initially designed as a bail-out temporary MCS, has evolved into a pivotal prophylactic tool for managing high-risk situations during cardiac procedures and heart transplantation (Figure 1). Its impact extends far beyond technological advancements, reflecting the imperative of ensuring equitable access to advanced medical treatments worldwide^[1]^.

In the United States of America, cardiac transplantation has emerged as the gold standard therapy for advanced heart failure. However, the limited availability of donors often leads to prolonged waiting periods for candidates, during which patients may experience hemodynamic deterioration. In such critical scenarios, IABP serves as a vital bridge to transplantation, often offering a sufficient, yet partial, circulatory assistance to patients while awaiting donor organs. Despite the emergence of alternative and more effective/powerful MCS devices, IABP still remains the initial preferred choice due to its lower invasiveness, cost-effectiveness, and superior safety profile^[2]^.

In recent years, doubts have surfaced regarding the efficacy of IABP, particularly in cases of myocardial infarction complicated by cardiogenic shock, as shown in the SHOCK-II Trial results, by Thiele et al.^[3]^. However, a reevaluation and appraisal of its role underscores its myriad indications in the perioperative phase of cardiac surgery. From mitigating post-cardiotomy shock to enhancing patient survival in high-risk cases, IABP continues to be a frontline approach in contemporary cardiac surgery, offering a balance between efficacy and safety^[4]^.

The management of cardiogenic shock remains a clinical challenge, with MCS emerging as a promising therapeutic avenue. However, the inconclusive evidence from randomized controlled trials and the predominance of alternative MCS devices present hurdles in the widespread adoption of IABP. Ongoing trials seek to address these gaps, shedding light on patient selection criteria and personalized treatment strategies tailored to individual patient needs^[5]^.

**Fig. 2:**
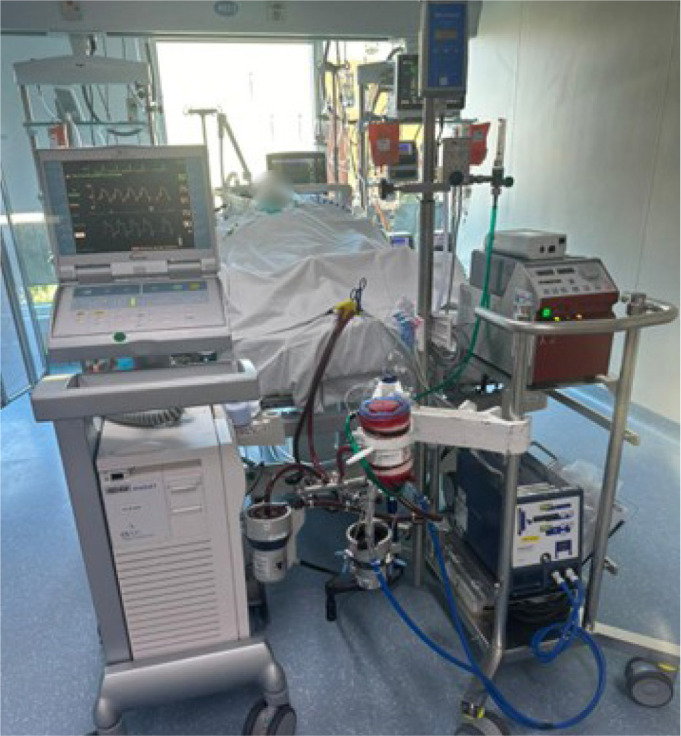
Patient in pre-transplantation status with intra-aortic balloon pump and veno-arterial extracorporeal membrane oxygenation support, Interagency Registry for Mechanically Assisted Circulatory Support (or INTERMACS) 1.

The evolution of organ allocation policies, exemplified by the United Network for Organ Sharing (or UNOS), reflects the dynamic nature of medical decision-making. The emphasis on granular listing criteria and improved risk stratification has led to a surge in the utilization of temporary MCS, notably IABP, as a bridge to transplantation (Figure 2). However, disparities in mortality risk among listed patients underscore the need for continued refinement of allocation algorithms to ensure equitable access to cardiac transplantation based on medical urgency^[6]^.

An analysis of data from the largest heart transplant center in Brazil offers valuable insights into the profound impact of IABP on patient outcomes. Over a series of ten years (2013 to 2024), 53.8% of the 528 consecutive heart transplants involved the use of IABP, further highlighting the importance of IABP in the transplantation process. With a significant proportion of transplant candidates relying on IABP as a bridge to transplantation, coupled with its low complication rates, its efficacy and safety profile remain unparalleled. Furthermore, the intra-axillary approach offers enhanced mobility, further underscoring its utility in resource-limited settings^[7]^.

Despite its established benefits, the utilization of IABP faces scrutiny in certain clinical scenarios, necessitating a nuanced approach to its application. While acknowledging its indispensable role in cardiac surgery and transplantation, ongoing research endeavors aim to elucidate its optimal use, particularly in the context of evolving treatment paradigms and patient-centered care^[8]^.

As we navigate the ever-evolving landscape of cardiac surgery and transplantation, IABP stands as a beacon of innovation and hope. Its transformative potential, especially in resource-constrained environments, underscores the imperative of equitable access to advanced medical therapies worldwide. By harnessing the power of technology and evidence-based practice, we can strive towards a future where every patient receives optimal care, irrespective of geographical boundaries or economic disparities. In summary, the editorial delves into the multifaceted role of IABP in reshaping the landscape of cardiac surgery and transplantation. Through a comprehensive analysis of real-world data and ongoing research endeavors, it advocates for a nuanced understanding of its efficacy and challenges, paving the way for informed decision-making and improved patient outcomes^[9]^.
